# Sociodemographic factors of violent deaths related to licit or ilicit psychoactive substances: a cross-sectional study, Ceará, Brazil, 2015-2019

**DOI:** 10.1590/S2237-96222024v33e2024152.en

**Published:** 2025-01-27

**Authors:** Wanderley Pinheiro de Holanda, Raimunda Hermelinda Maia Maceno, Maria Augusta Drago Ferreira

**Affiliations:** 1 Perícia Forense do Ceará, Núcleo de Toxicologia Forense, Fortaleza, CE, Brazil; 2 Universidade Federal do Ceará, Departamento de Fisioterapia, Fortaleza, CE, Brazil; 3 Universidade Federal do Ceará, Departamento de Farmácia, Fortaleza, CE, Brazil

**Keywords:** Violencia, Toxicología Forense, Medicina Legal, Drogas de Abuso, Mortalidad, Estudio Transversal, Violence, Forensic Toxicology, Forensic Medicine, Illicit Drugs, Mortality, Cross-Sectional Study

## Abstract

**Objective:**

To assess association between sociodemographic factors, presence of licit or illicit psychoactive substances, and types of legal death in Ceará state, Brazil.

**Methods:**

Cross-sectional study based on autopsy data and toxicology tests on victims of cases registered by the Ceará Forensic Expert service, from 2015 to 2019.

**Results:**

Of the 4,198 cases analyzed, 55.6% were positive for licit and/or illicit substances, with greater frequency of benzodiazepines (22.4%) and cocaine (21.7%) among males (45.0%), young adults (58.7%), single people (49.0%), and those with low education levels (52.8%). Association was found between benzodiazepines (29.5%) and tricyclic antidepressants (15.6%) and suicides; cocaine (28.1%) and cannabis (27.1%) and homicides; and cocaine (28.2%) and tricyclic antidepressants (5.9%) and suspicious deaths.

**Conclusion:**

Sociodemographic factors were associated with the use of psychoactive substances and types of legal death. Benzodiazepines and cocaine were the most frequent substances in suicide and homicide cases, respectively.

## Study contributions

**Main results:**

Cocaine and cannabis were associated with greater risk of homicide, while benzodiazepines and tricyclic antidepressants were associated with greater risk of suicide.

**Implications for services:**

The study emphasizes the need for drug use preven-tion policies, as well as treatment and reintegration of drug users, and safety measures to reduce violence, aimed at public health and social justice.

**Perspectives:**

Further studies are needed to assess causality be-tween psychoactive substances and violent deaths.

## RESUMEN

**Objetivo:**

Evaluar la relación entre factores sociodemográficos, presencia de sustancias psicoactivas lícitas y/o ilícitas y las muertes legales en Ceará.

**Métodos:**

Estudo transversal realizado com base em dados de necropsias y exámenes toxicológicos de casos registrados por el Peritaje Forense de Ceará, entre 2015 y 2019.

**Resultados:**

Se evaluaron 4.198 casos, el 55,6% resultaron positivos para sustancias lícitas y/o ilícitas, con mayor frecuencia de benzodiazepinas (22,4%) y cocaína (21,7%) entre hombres (45,0%), adultos jóvenes (58,7%), solteros (49,0%) y baja escolaridad (52,8%). Las benzodiazepinas (29,5%) y los antidepresivos tricíclicos (15,6%) se asociaron a suicidios, la cocaína (28,1%) y marihuana (27,1%) a homicidios; la cocaína (28,2%) y antidepresivos tricíclicos (5,9%) a muertes sospechosas.

**Conclusión:**

Los factores sociodemográficos se asociaron con el uso de sustancias psicoactivas con tipos de muerte legal. Las benzodiazepinas y la cocaína fueron las sustancias más comunes en los casos de suicidio y homicidio, respectivamente.

## INTRODUCTION

Violence is a global problem and can be associated with use of psychoactive substances.^1^ Its causes, influenced by multiple factors, are complex and not fully understood.^1,2^ Public health, using scientific methods, seeks to prevent violence through accumulation of knowledge on the issue.^3^

Knowing the circumstances and characteristics of deaths and chemical substances related to them is important for a better assessment of the panorama of victim mortality and, therefore, enabling application of more efficient policies and resources to reduce this form of mortality.^4^

Analysis of mortality and knowledge of the epidemiological profile of victims involved in cases of use of one or multiple chemical agents, as well as identification of health conditions and the most susceptible groups, are of fundamental importance for a health system to take action and develop specific policies.^2^ However, research relating the use of specific substances and the risk factors that lead to death is still limited.^4^

Therefore, it must be considered that, in addition to identifying the chemical substances involved in these deaths, it is necessary to understand how the pattern of violent mortality is related to the substances themselves.^4,5^ Therefore, more research is essential to expand understanding and develop more effective prevention and intervention strategies.^5^

 Violence associated with use of psychoactive substances requires attention and continuous studies. Previous research has already demonstrated this association.^4-7^ Therefore, the relevance of this study lies in verifying the relationship between use of psychoactive substances and mortality from violent causes in the state of Ceará. Understanding this scenario is crucial for public health and security, as it enables the mortality scenario to be assessed and more efficient resources and public policies targeted towards reducing violence.^4,5^

In this sense, forensic medicine and forensic toxicology play a crucial role by providing detailed information about the circumstances of deaths. Furthermore, they help to establish cause and effect relationships between chemical agents and violent deaths. As part of public security services, they are responsible for conducting forensic investigations and determining the cause of death in cases of homicides, suicides, accidental deaths or other unnatural or suspicious deaths.^7-9^

The objective of this study was to assess association between sociodemographic factors, presence of licit or illicit psychoactive substances, and types of legal death in Ceará state, Brazil.

## METHODS

Cross-sectional study, using deterministic and probabilistic record linkage techniques, of cases of violent deaths, with presumed involvement of legal and/or illicit chemical substances, recorded by the Ceará Forensic Expert Service (*Perícia Forense do Ceará*), from 2015 to 2019.

Brought into being by State Law No. 14055/2008, the Ceará Forensic Expert Service is based in the city of Fortaleza and is a technical-scientific body subordinated to the Public Security Department, which operates throughout the state of Ceará, and the attribution of which is the production of evidence of a scientific nature in criminal investigations.^9^

The data on the cases analyzed in the present study came from autopsy exams carried out at the Forensic Thanatology Center of the Forensic Medicine Service (*Coordenadoria de Medicina Legal*), and from the results of toxicology screening analyses performed at the Forensic Toxicology Center (*Núcleo de Toxicologia Forense*) of the Forensic Laboratory Analysis Service (*Coordenadoria de Análises Laboratoriais Forenses*), both being part of the Ceará Forensic Expert Service.

Cases that had toxicology screening analysis performed on whole blood samples were included in the study. Cases in which records indicated bodies in a state of putrefaction or charred, situations in which blood samples from the victim were not available and cases resulting from exposure to pesticides, ethyl alcohol and volatile substances were excluded.

Death data were collected from three recording systems: the Integrated Management System (*Sistema de Gestão Integrada*), which operated until 2019; the Galileu System for forensic expert service management implemented in 2019; and the automated Evidence® system (Randox Laboratories Ltd.), which stores the results of toxicology screening analyses obtained using the immunoassay technique.

Data linkage was performed using deterministic, probabilistic and join record linkage.^10^

Deterministic linkage is done through a unique identification field or data (id) common to both databases that can be linked.^10^ Probabilistic linkage was used to data that refer to the same entity, even if they do not have unique identifiers. Scores were assigned based on the similarity between the values of the potential identifiers.^11^

We used Stata® (Statistics/Data Analysis) BE version 17 software to perform linkages and statistical calculations. In the deterministic linkage we used the “merge” and “merge joinby pairwise” functions. The unique identifiers (id) we used were the request number and the combination of entities: agency code, file number, file year, record number, record year and forensic center. 

In the probabilistic linkage, we used the “dtalink” function to match identifiers, assigning scores to assess the probability of correspondence between records as follows: name, year of the file (+10 for coincidences; -5 for non-coincidences) and input date (+5 for exact days; -6 for differences greater than 30 days). 

In order to obtain sensitivity and specificity when preparing probability calculations, a criterion was established whereby the sum of the scores for the combination of pairs had to be greater than or equal to 34.^12^ After this step, the consistency of the pair formed was assessed to determine whether it would be accepted or rejected. 

The independent variables were categorized into sex (male/female); age (≤ 14 years; 15-29 years; 30-49 years; and ≥ 50 years); marital status (single, separated/divorced/widowed, married/civil partnership); level of schooling (illiterate, literate, complete elementary education, complete high school education, higher education); occupation (students, workers in the agricultural/industrial/services/commerce sectors, civil service/sciences, retired/unemployed); legal death (homicides, suicides, accidental deaths [including road traffic accidents], suspicious deaths [including undetermined deaths], other forms of death [drowning, electrocution etc.]; instrument/manner of death. The outcome was detection of illicit substances and/or drugs in toxicology tests. In other words, a sample was considered positive (detected) when at least one of the substances studied presented a concentration equal to or greater than the cutoff value in nanograms per deciliter (ng/mL). 

The cutoff values for illicit substances were: methamphetamine ≥ 110 ng/mL; amphetamine ≥ 100 ng/mL; 3-4, methylenedioxymethamphetamine (MDMA, ecstasy) and cocaine/benzoylecgonine ≥ 50 ng/mL; delta-9-THC (cannabis) ≥ 10 ng/mL; phencyclidine (PCP) ≥ 5 ng/mL. For licit drugs/drug classes were: tricyclic antidepressants ≥ 60 ng/mL; barbiturates ≥ 50 ng/mL; benzodiazepines, buprenorphine, methadone, oxycodone, opioids, meprobamate and dextromethorphan ≥ 10 ng/mL; opiates ≥ 25 ng/mL; tramadol ≥ 5 ng/mL; and fentanyl ≥ 2 ng/mL.

Shapiro-Wilk statistical tests were applied to verify the normality of data distribution. Each prevalence ratio (PR) was calculated by dividing the prevalence of the event (presence of the psychoactive substance) in the group with drug-related deaths by the prevalence of the same event in a reference group. Fisher’s exact test (two-sided p-value) and the 95% confidence interval (95%CI) were calculated to assess the significance of this relationship. Results with p-value < 0.05 were considered statistically significant. 

The project was approved by the Research Ethics Committee of the *Universidade Federal do Ceará*, Opinion No. 5,192,820, in 1/5/2022, certificate of submission for ethical appraisal No. 53707521.4.0000.5054.

## RESULTS

From 2015 to 2019, 5,329 deaths suspected of involving psychoactive chemical substances were recorded. In 4,400 cases, toxicology screening tests were carried out to detect drugs and medications. After excluding 202 records, 4,198 cases were included, accounting for 12.0% of the 34,304 autopsy exams performed. 

Of the 4,198 cases examined, 55.6% were positive for licit and/or illicit substances and 35.9% were positive for illicit substances. The majority of individuals were male (81.7%), aged between 30 and 49 years old (41.3%) or between 15 and 29 years old (27.2%), single (67.4%), literate (59.6%) and workers in the agriculture, industry, services and commerce sectors (68.4%). The highest frequency of deaths occurred in hospitals (37.1%), with the majority of them classified as suspicious deaths (53.9%) (Table 1). The drugs/drug classes with the highest detection, excluding cases originating in healthcare establishments, were benzodiazepines (22.4%). Among illicit substances, there was greater detection of cocaine (21.7%), followed by cannabis (11.6%) (Table 2).

**Table 1:** Characteristics of cases with *post mortem* toxicology tests performed, Ceará, Brasil, 2015-2019

**Sociodemographic characteristics**	**n**	**%**
**Sex**	2,347	100.0
Male	1,918	81.7
Female	429	18.3
**Age group (years)**	31	1.4
0-4	28	1.3
5-14	601	27.2
15-29	913	41.3
30-49	298	13.5
50-59	178	8
60-69	163	7.4
> 69	163	7,4
**Marital status**	1,855	100.0
Single	1,250	67.4
Married/civil partnership/widowed/	436	23.5
Separated/divorced	169	9.1
**Level of schooling**	1,769	100.0
Illiterate	172	9.7
Literate	1,054	59.6
Complete elementary education	127	7.2
Complete high school education	296	16.7
Complete higher education	83	4.7
Not informed	37	2.1
**Occupation**	1,284	100.0
Retired/unemployed/student	265	20.6
Agriculture, industry, services and commerce	878	68.4
Civil service/sciences	141	11
**Place of death**	1,215	100.0
Hospital/Emergency Care Unit	450	37.1
Rural area/public thoroughfare	175	14.4
Home/prison unit	148	13
Unclassified address	443	36.5
**Type of death**	2,030	100.0
Suspicious	1,095	53.9
Accidental	438	21.6
Suicide	237	11.7
Homicide	203	10
Outros	57	2.8
**Manner of death**	1,609	100.0
Drowning	59	3.7
Cold weapon	6	0.4
Electric shock	14	0.9
Hanging	55	3.4
Poisoning	223	13.9
Firearm projectile	200	12.4
Fall on same level	63	3.9
Fall	6	0.4
Other	983	61.1

**Table 2:** Frequency of substances detected in cases of violent deaths, Ceará, Brazil, 2015-2019

Licit drugs/drug classes detected	All cases			Hospital origin excluded		
	n	Positive	%	n	Positive	%
Benzodiazepines	4,198	1,074	25.6	3,748	841	22.4
Meprobamate	357	26	7.3	269	19	7.1
Opioids	357	24	6.3	269	16	5.9
Tricyclic antidepressants	4,198	190	4.5	4,163	155	3.7
Barbiturates	4,141	149	3.6	4,141	149	3.6
Oxycodone	357	12	3.4	269	9	3.3
Fentanyl	357	29	8.7	269	7	2.8
Tramadol	357	12	3.4	269	5	1.9
Opiates	4,137	100	2.4	3,694	64	1.7
Methadone	4,125	38	0.9	3,684	27	0.7
Zolpidem	357	2	0.6	269	2	0.7
Buprenorphine	4,198	8	0.2	3,748	8	0.2
**Illicit substances**						
Cocaine	4,198	912	21.7			
Cannabis	4,198	488	11.6			
Methamphetamine	4,198	292	7.0			
3-4, methylenedioxymethamphetamine (MDMA, ecstasy)	3,840	127	3.3			
Phencyclidine	4,198	37	0.9			

We found that a single substance, licit and/or illicit, was detected in 29.1% of cases. For illicit substances alone, single substances accounted for 23.1%. Detection of multiple substances showed greater association between illicit substances (12.8%) than between licit substances (8.2%).

Taking the year 2015 as a basis, in 2018 there was a significant detection of psychoactive substances (48.9%), illicit substances (42.9%) and amphetamine compounds (21.6%) (Table 3). Men (45.0%) and young people aged 15-29 (32.0%) led the prevalence rates, for both psychoactive and illicit substances. Single people (49.0%) accounted for higher prevalence of detection of psychoactive substances than those who were married/living in a civil partnership. Individuals with complete elementary education (52.8%) had higher prevalence of psychoactive substances, compared to those who were illiterate. When examining occupations, students stood out (55.5%). 

**Table 3:** Prevalence, prevalence ratio (PR) and confidence interval (95%CI) of the presence of licit and illicit psychoactive substances, Ceará, Brazil, 2015-2019

		Variables	Licit and illicit substances			Illicit substances			Amphetamine compounds		
n	Positive (%)		PR (95%CI)	p-value	Positive (%)	PR (95%CI)	p-value	n	Positive (%)	PR (95%CI)	p-value
Year	4,198							3,840			
2015	1,511	533 (35.3)	1.00		461 (30.5)	1.00		1,511	115 (7.6)	1.00	
2016	963	360 (37.4)	1.06 (0.95;1.18)	0.287	312 (32.4)	1.06 (0.94;1.19)	0.323	960	74 (7.7)	1.01 (0.76;1.34)	0.93
2017	625	262 (41.9)	1.19 (1.06;1.33)	0.004	219 (35.0)	1.14 (1.01;1.31)	0.041	622	122 (19.6)	2.58 (2.03;3.27)	<0.001
2018	662	324 (48.9)	1.39 (1.25;1.54)	<0.001	284 (42.9)	1.41 (1.25;1.58)	<0.001	645	139 (21.6)	2.83 (2.25;3.56)	<0.001
2019	437	190 (43.5)	1.23 (1.09;1.40)	0.002	112 (25.6)	0.84 (0.70;1.0)	0.049	102	9 (8.8)	1.16 (0.61;2.22)	1.17
**Sex**	2,347							2,001			
Male	1,918	864 (45)	1.11 (1.02;1.32)	<0.001	733 (38.2)	1.23 (1.15;1.31)	<0.001	1,634	232 (14.2)	1.00	
Female	429	167 (38.9)	1.0		104 (24.2)	1.00		367	68 (18.5)	1.30 (1.02;1.66)	0.04
**Age group (years)**	2,213							1,877			
0-14	60	14 (23.3)	1.00		9 (15.0)	1.00		51	6 (11.8)	0.91 (0.41;1.98)	0.82
15-29	601	353 (58.7)	2.52 (1.58;4.00)	<0.001	322 (53.6)	3.57 (1.95;6.55)	<0.001	531	69 (13.0)	1.00	
30-49	913	434 (47.5)	2.04 (1.28;3.24)	<0.001	347 (38.0)	2.53 (1.38;4.65)	<0.001	776	122 (15.7)	1.21 (0.92;1.59)	0.171
> 49	639	172 (26.9)	1.15 (0.72;1.86)	0.549	109 (17.1)	1.13 (0.61;2.12)	0.684	519	93 (17.9)	1.38 (1.03;1.84)	0.03
**Marital status**	1,855							1,528			
Single	1,250	613 (49)	1.43 (1.25;1.65)	<0.001	515 (41.2)	1.83 (1.52;2.21)	<0.001	1,037	162 (15.6)	1.07 (0.81;1.40)	0.726
Separated/divorced/widowed	169	60 (35.5)	1.04 (0.82;1.32)	0.758	49 (29.0)	1.29 (0.96;1.73)	0.094	134	27 (20.1)	1.05 (0.79;1.40)	0.158
Married/civil partnership	436	149 (34.2)	1.00		98 (22.5)	1.00		357	53 (14.8)	1.00	
**Level of schooling**	1,732							1,412			
Illiterate	172	57 (33.1)	1.00		39 (22.7)	1.00		137	25 (18.2)	1.27 (0.86;1.88)	0.237
Literate	1,054	478 (45.4)	1.37 (1.09;1.7)	0.054	391 (37.1)	1.64 (1.23;2.18)	<.001	877	126 (14.4)	1.00	
Complete elementary educa-tion	127	67 (52.8)	1.60 (1.22;2.08)	0.003	58 (45.7)	2.01 (1.44;2.81)	<0.001	96	14 (14.6)	1.02 (0.61;1.69)	0.954
Complete high school educa-tion	296	138 (46.6)	1.40 (1.10;1.79)	0.033	116 (39.2)	1.72 (1.27;2.35)	<0.001	238	42 (17.6)	1.23 (0.89;1.69)	0.21
Higher education	83	32 (38.6)	1.16 (0.82;1.64	0.657	21 (25.3)	1.11 (0.70;1.77)	0.624	64	11 (17.2)	1.2 (0.68;2.10)	0.538
**Occupation**	1,263							1,144			
Student	137	76 (55.5)	1.71 (1.28;2.35	<0.001	65 (47.4)	1.82 (1.28;2.58)	0.005	127	19 (15.0)	0.99 (0.63;1.54)	0.959
Agriculture, industry, services and commerce	893	402 (45.0)	1.41 (1.07;1.85	0.008	338 (37.8)	1.45 (1.06;1.99)	0.013	806	122 (15.1)	1.00	
Civil service	114	49 (43.0)	1.34 (0.96;1.88	0.073	40 (35.1)	1.34 (0.91;1.99)	0.135	102	19 (18.6)	1.23 (0.79;1.91)	0.360
Retired/unemployed	119	38 (31.9)	1.00		31 (26.1)	1.00		109	26 (23.9)	1.58 (1.09;2.29)	0.02

Taking the year 2015 as a basis, in 2018 there were significant differences in detection of cocaine (27.0%) and MDMA (9.6%). Cannabis (14.1%) had a greater share in 2016 compared to 2019. Regarding sex, males had significantly higher prevalence rates for cocaine (26.5%) and cannabis (12.9%). In the 15-29 age group, there was higher detection of cocaine (38.1%) and cannabis (26.0%). When examining occupations, taking retired/unemployed people as a basis, workers in the public sector and the sciences (31.4%), the agricultural, industrial and services sectors (21.9%) and students (7.60%) showed high prevalence for cocaine, while students had higher prevalence for cannabis (21.2%) (Table 4).

**Table 4:** Prevalence, prevalence ratio (PR) and confidence interval (95%CI) of the presence of cocaine, cannabis and ecstasy, Ceará, Brazil, 2015-2019

Variable		Cocaine			Cannabis				Ecstasy		
	n	Positive (%)	PR (95%CI)	p-value	Positive (%)	PR (95%CI)	p-value	n	Positive (%)	PR (95%CI)	p-value
Year	4,198							3,840			
2015	1,511	302 (20.0)	1.00		202 (13.4)	2.54 (1.67;3.85)	<0.001	1,511	8 (0.5)	1.00	
2016	963	212 (22.0)	1.10 (0.94;1.29)	0.226	136 (14.1)	2.68 (1.75;4.11)	<0.001	960	17 (1.8)	3.34 (1.44;7.71)	0.005
2017	625	124 (19.8)	0.99 (0.82;1.19)	0.938	50 (8.0 )	1.52 (0.94;2.45)	0.085	622	38 (6.1)	11.5 (5.41;24.59)	<0.001
2018	662	179 (27.0)	1.35 (1.15;1.59)	<0.001	77 (11.6)	2.21 (1.41;3.46)	<0.001	645	62 (9.6)	18.1 (8.74;37.69)	<0.001
2019	437	95 (21.7)	1.09 (0.87;1.33)	0.423	23 (5.3)	1.00		102	2 (2.0)	3.70 (0.79;17.21)	0.097
**Sex**	2,347							2,001			
Male	1,918	509 (26.5)	2.27 (1.74;2.98)	<0.001	247 (12.9)	3.06 (1.92;4.89)	<0.001	1,634	73 (4.5)	1.00	0.08
Female	429	50 (11.7)	1.00		18 (4.2)	1.00		367	25 (6.8)	1.52 (0.98;2.34)	
**Age group (years)**	2,213							1,877			
0-14	60	3 (5.0)	0.88 (0.28;2.79)	0.083	3 (5.0)	1.88 (0.57;6.23)	0.307	51	4 (7.8)	2.60 (0.90;7.49)	0.082
15-29	601	229 (38.1)	6.76 (4.85;9.44)	<0.001	156 (26.0)	9.75 (5.99;15.89)	<0.001	531	16 (3.0)	1.00	
30-49	913	253 (27.7)	4.91 (3.52;6.87)	<0.001	69 (7.6)	2.84 (1.69;4.78)	<0.001	776	38 (4.9)	1.62 (0.91;2.88)	0.096
> 49	639	36 (5.6)	1.00		17 (2.7)	1.00		519	37 (7.1)	2.36 (1.33;4.19)	0.003
**Marital status**	1,855							1,528			
Single	1,250	357 (28.6)	2.04 (1.60;2.61)	<0.001	169 (13.5)	3.1 (1.95;4.92)	<0.001	1,037	61 (5.9)	1.31 (0.77;2.24)	0.679
Separated/divorced/widowed	169	29 (17.2)	1.23 (0.82;1.84)	0.327	7 (4.1)	0.95 (0.41;2.22)	0.907	134	8 (6.0)	1.33 (0.58;3.04)	0.497
Married/civil partnership	436	61 (14.0)	1.00		19 (4.4)	1.00		357	16 (4.5)	1.00	
**Level of schooling**	1,732							1,412			
Illiterate	172	17 (9.9)	0.68 (0.34;1.36)	0.284	12 (7.0)	0.96 (0.37;2.48)	0.25	137	11 (8.0)	1.60 (0.84;3.28)	0.350
Literate	1,054	279 (26.5)	1.83 (1.07;3.11)	0.018	120 (11.4)	1.57 (0.71;3.47)	0.941	877	44 (5.0)	1.00	
Complete elementary education	127	45 (35.4)	2.45 (1.38;4.35)	0.001	18 (14.2)	1.96 (0.81;4.73)	0.129	96	4 (4.2)	0.83 (0.30;2.26)	0.534
Complete high school education	296	80 (27.0)	1.86 (1.07;3.25)	0.02	28 (9.5)	1.3 (0.56;3.05)	0.531	238	15 (6.3)	1.25 (0.71;2.21)	0.308
Higher education	83	12 (14.5)	1.00		6 (7.2)	1.00		64	6 (9.4)	1.86 (0.82;4.21)	0.456
**Occupation**	1,263							1,144			
Student	137	9 (7.6)	4.15 (2.11;8.15)	<0.001	29 (21.2)	6.03 (2.18;16.65)	<0.001	127	7 (5.5)	1.13 (0.52;2.49)	0.745
Agriculture, industry, services, commerce	893	25 (21.9)	3.54 (1.87;6.69)	0.003	99 (11.1)	3.16 (1.18;8.42)	<0.001	806	39 (4.8)	1.00	
Civil service	114	43 (31.4)	2.90 (1.41;5.94)	<0.001	4 (3.5)	1.00		102	8 (7.8)	1.62 (0.77;3.37)	0.201
Retired/unemployed	119		1.00		2 (1.7)	0.48 (0.09;2.56)	0.389	109	11 (10.0)	2.08 (1.10;3.94)	0.027

Table 5 shows the prevalence of licit and illicit substances in different types of death in relation to accidental deaths. The results show that, regarding deaths due to suicide, there was a higher prevalence of licit substances (52.3%), benzodiazepines (29.5%) and tricyclic antidepressants (15.6%). In the case of suspicious deaths, 45.3% tested positive for licit substances, with 15.9% for benzodiazepines and 5.9% for tricyclic antidepressants. Deaths related to detection of illicit substances were significantly frequent in cases of homicides (51.7%), followed by suspicious deaths (39.8%). Cocaine (28.2%) had a significant participation in suspicious deaths, while in homicides cocaine (28.2%), followed by cannabis (27.1%), were the most prevalent substances among the drugs studied.

**Table 5:** Prevalence, prevalence ratio (PR) and confidence interval (95%CI) of the presence of licit and illicit psychoactive substances with type of death, Ceará, Brazil, 2015-2019

**Types of death**		**Licit substances**			**Benzodiazepines**			**Tricyclic antidepressants**		
	**n**	**Positive (%)**	**PR (95%CI)**	**p-value**	**Positive (%)**	**PR (95%CI)**	**p-value**	**Positive (%)**	**PR (95%CI)**	**p-value**
Accidental	438	155 (35.4)	1.00		59 (13.5)	1.00		13 (3.0)	1.00	
Homicide	203	67 (33.0)	0.93 (0.74;1.18)	0.555	29 (14.3)	0.93 (0.62;1.40)	0.730	3 (1.5)	0.50 (0.14;1.73)	0.260
Suicide	237	124 (52.3)	1.48 (1.24;1.76)	<0.001	70 (29.5)	2.11 (1.56;2.84)	<0.001	37 (15.6)	5.26 (2.85;9.70)	<0.001
Suspicious	1,094	496 (45.3)	1.28 (1.11;1.48)	<0.001	174 (15.9)	1.22 (0.94;1.60)	0.133	65 (5.9)	2.00 (1.12;3.59)	<0.001
Other	56	28 (50.0)	1.41 (1.06;1.89)	0.033	12 (21.4)	1.54 (0.90;2.64)	0.127	3 (5.4)	1.80 (0.53;6.14)	0.341
**Types of death**		**Illicit substances**			**Cocaine**			**Cannabis**		
	**n**	**Positive**	**(%)**	**PR**	**Positive**	**(%)**	**PR**	**Positive**	**(%)**	**PR**
Accidental	438	103 (23.5)	1.00		65 (14.8)	1.00		31 (7.1)	1.00	
Homicide	203	105 (51.7)	2.20 (1.77;2.73)	<0.001	57 (28.1)	1.89 (1.38;2.59)	<0.001	55 (27.1)	3.83 (2.55;5.75)	<0.001
Suicide	237	61 (25.7)	1.09 (0.83;1.44)	0.521	37 (15.6)	1.05 (0.73;1.53)	0.789	13 (5.5)	0.78 (0.41;1.45)	0.424
Suspicious	1,094	435 (39.8)	1.69 (1.41;2.03)	<0.001	308 (28.2)	1.90 (1.49;2.42)	<0.001	111 (10.1)	1.43 (0.98;2.10)	0.061
Other	56	16 (28.6)	1.21 (0.78;1.90)	0.405	11 (19.6)	1.32 (0.74;2.35)	0.348	4 (7.1)	1.01 (0.37;2.75)	0.985

## DISCUSSION

Between 2015 and 2019, we found that prevalence of deaths with detection of at least one psychoactive substance was high. The most frequent substances detected by toxicology tests were benzodiazepines and cocaine. The majority of individuals involved in these cases were male, young adults, single, with a basic level of literacy and worked in activities related to the agricultural, industrial, services and commerce sectors. Suspicious/undetermined types of death predominated, while predominant causes of death were poisoning and firearm projectile. The most prevalent illicit drugs among young male adults were cocaine and cannabis; amphetamine compounds were more frequent among females. Cocaine was linked to suspicious/undetermined deaths, and both cannabis and cocaine were linked to homicide cases.

In the cases of deaths assessed, the majority of affected individuals were male, young adults, single, with a low level of education. These findings corroborate results from similar studies. Research carried out in Warri, Nigeria, in 2020,^13^ based on necroscopic examinations, revealed that males (90.5%) and young adults between 21 and 30 years old (39.8%) were the most involved in these incidents. In a forensic medicine context, drug-related deaths were assessed in a study carried out in Atacama, Chile, between 2010 and 2018, revealing that individuals involved were male in 80.0% of cases.^14^ A study conducted in 2016,^15^ reported that in the cities of Joinville-SC and Maceió-AL, in Brazil, violent deaths associated with cocaine and cocaethylene occurred mainly among males and individuals in the 19-50 age group.

In our study, the toxicology tests showed the presence of at least one psychoactive substance in more than half of the cases of violent death assessed. Illicit drugs were detected in more than a third of these cases. A study carried out in, Brasília, Brazil, between 2006 and 2008,^16^ reported that at least one substance (drug or other toxic agent) was detected in 21.7% of the cases assessed, more frequently among young men, single men and men with a low level of schooling. Furthermore, another study, conducted in Parma, Italy, between 2009 and 2016, on forensic medicine investigations, revealed that 71.8% of men had positive toxicology test results.^17^

The tests examined in our study revealed that drugs from the benzodiazepine class and illicit drugs (cocaine and cannabis) were detected more frequently in young adult males. Regarding drugs and/or drug classes, we found high prevalence of benzodiazepines, followed by amphetamine compounds, meprobamate and opioids/opiates. According to a study,^18^ conducted in 2015, in Brazil, the most consumed medications that had not been prescribed or were different from the treatment indicated were benzodiazepines (3.9%), opiates (2.9%) and amphetamines (1.4%). Although medications bring significant benefits, inappropriate use can result in dependence, and they can even be used in the perpetration of crimes.

Previous studies indicate the indiscriminate use of benzodiazepines.^19-21^ Despite having low toxicity, they can cause serious adverse effects when used inappropriately or in combination with other substances.^22^ A study carried out at the Toxicological Information and Assistance Center, in Fortaleza, Ceará, Brazil, in 2013, showed that benzodiazepines were included among the main classes of drugs involved in cases of severe acute poisoning, sometimes fatal, resulting from suicide attempts.^23^

Given that they act as central nervous system depressants, benzodiazepines have been put in the food or drink of victims, with the intention of committing crimes against them, such as in cases of rape.^8^ For this reason, indiscriminate use has been associated with violence, crime and/or mortality.^8,19,22^

Violence can be perpetrated in a variety of ways,^24^ and varies depending on the sex of the victim. Among males, the most common causes of violent death are assaults and traffic accidents,^25^ while among females suicide is more prevalent.^26^ There is a strong relationship between use of psychoactive substances, licit and/or illicit, and the form of violence committed. This association has effects on different populations, be it in the form of victimization or perpetration of other crimes.^27^ As stated above, our study revealed that the types of psychoactive substances and their combinations varied according to age, sex and type of violent death. As such, among illicit substances, cocaine and cannabis were detected more frequently in male individuals. On the other hand, amphetamine compounds (methamphetamine and ecstasy) were present in greater proportions in females.

Licit substances had greater participation in suicide cases, followed by suspicious/undetermined deaths. Benzodiazepines and tricyclic antidepressants were the most involved in suicide deaths. In homicide cases, at least one illicit substance was present in the majority of victims, with greater involvement of cocaine and cannabis.

Studies conducted in South America, North America and Australia, between 2009 and 2021, pointed to the significant share of benzodiazepines, alone or in combination with other substances, as being responsible for acute exogenous poisonings, often resulting from suicide attempts or suicide itself.^4,6,22,23^ In 2016, a study^4^ in Florida/USA, reported that central nervous system depressant substances, such as benzodiazepines, meprobamate (a metabolite of carisoprodol), opiates and zolpidem, are more associated with accidental deaths and/or suicides. In 2009, a study carried out in Australia investigated the presence of psychoactive substances in cases of death due to homicide and suicide, and the presence of psychoactive substances was detected in 65.5% of cases; multiple drugs in 25.8%; illicit substances in 23.9%; and various illicit drugs in 5.3% of cases.^6^ In turn, another article^22^ reported that, in Hungary, between 1990 and 2001, selective serotonin reuptake inhibitors, barbiturates and benzodiazepines were the main substances causing deaths due to overdose in 79.0% of suicide cases.

The results obtained in our study indicated a trend of increasing association between illicit drugs and violent deaths, especially in the case of cocaine and cannabis. In cases of homicide, the victims who used illicit drugs were, for the most part, male, young adults, and the substances detected most frequently were cocaine and cannabis. As in cases of suspicious/undetermined death, cocaine was the most frequently detected substance. Researchs conducted in Chile,^14^ between 2010 and 2018, and in Brazil,^15^ between 2013 and 2015, demonstrated that substances associated with homicides were mainly cocaine and cannabis.

In Brazil, a study conducted in São Paulo, in 2019, showed the majority of victims who used cannabis and cocaine were male (82.2%) and over 30 years old (63.0%).^7^ In another study carried out in Vitória, in 2015, the authors analyzed 150 victims of homicides and found that 47.3% of them tested positive for cannabis, 36.7% for cocaine, and 20.6% for both substances.^28^ In Belo Horizonte, investigating association between drugs (ethanol and others) and homicide, from 2000 to 2009, the presence of cocaine and cannabis was detected in 25.1% of victims. ^29^

In our study, victims with a positive toxicology test result had a sociodemographic profile similar to that of drug users undergoing treatment at the Alcohol and Drugs Psychosocial Care Center (*Centro de Atenção Psicossocial – Álcool e Drogas*) in Fortaleza, in 2018. This profile includes individuals who started using psychoactive substances when they were adolescents (63.6%), predominantly male (84.0%), with an average age of 38.9 years, low education (38.2%); and single, separated or divorced people accounting for 53.4% of records.^30^ Deaths, especially among young male adults, may be related to the effects of psychoactive substances, such as the disinhibition caused by ethanol and/or paranoid episodes by cocaine, that can result in violent behavior. Furthermore, these deaths may be related to exposure to violent places to buy drugs and to participation in criminal activities.^7^

It is a cross-sectional study based on secondary data, thus preventing inference of causality. Although associations between the presence of psychoactive substances and violent deaths are identifiable, it cannot be said that one causes the other. Some statistical techniques, such as regression models with control variables, were used to minimize confounding factors and strengthen the relationships found. 

The positive results as to the presence of psychoactive substances in the samples analyzed were based on toxicology screening tests, of a presumptive nature. These tests require confirmation through additional analytical techniques, such as gas or liquid chromatography-mass spectrometry. Lack of confirmation can lead to false positive results. The data relating to the toxicology tests were treated with caution and interpreted in light of the set of information available. We highlight the importance of carrying out confirmatory analyses in future studies, which can contribute to the robustness of their conclusions.

The incompleteness of the data in the original records and the changes made to record system during the study period indicate a compromise with the quality and representativeness of the information collected. We made efforts to ensure data consistency and integrity by cross-checking and correcting inconsistencies whenever possible.

The results of this study demonstrated significant association between different types of legal death, demographic characteristics and toxicological profile, related to psychoactive substances in the state of Ceará. The data point to the prevalence of licit and/or illicit substances among young men, single men and those with low education levels. The distribution of these substances in the total number of related deaths showed frequent presence of benzodiazepines and cocaine. Furthermore, association between illicit drugs and homicide deaths was found, especially for cocaine and cannabis, while benzodiazepines and tricyclic antidepressants were strongly associated with suicide deaths. 

The relationship between the types of legal death and the use of psychoactive substances indicates a significant contribution to years of life lost due to premature death. Identifying the profile of populations associated with these substances is crucial for the development of effective public policies to prevent and mitigate violent deaths.

## References

[1] ONUDC (2020). World Drug Report 2020.

[2] Bochner R, Freire MM (2020). Análise dos óbitos decorrentes de intoxicação ocorridos no Brasil de 2010 a 2015 com base no Sistema de Informação sobre Mortalidade (SIM). Ciênc Saúde Colet.

[3] Dahlberg LL, Krug EG (2006). Violência: um problema global de saúde pública. Ciência & Saúde Coletiva.

[4] Lee D, Delcher C, Maldonado-Molina MM, Thogmartin JR, Goldberger BA (2016). Manners of Death in Drug-Related Fatalities in Florida. Journal of Forensic Sciences.

[5] Lemos YV, Wainstein AJA, Savoi LM, Drummond-Lage AP (2019). Epidemiological and toxicological profile of homicide victims in a legal medicine unit in Brazil. J Forensic Leg Med.

[6] Darke S, Duflou J, Torok M (2009). Drugs and violent death: Comparative toxicology of homicide and non-substance toxicity suicide victims. Addiction.

[7] Takitane J (2019). Drogas de abuso em vítimas de mortes violentas no município de São Paulo.

[8] Dorta DJ, Costa JL da, Yonamine M, Martinis BSD (2018). Toxicologia Forense.

[9] PEFOCE (2018). Medicina Legal: a mais antiga perícia desenvolvida no Ceará.

[10] Gu L, Baxter R, Vickers D, Rainsford C (2003). Record Linkage: Current Practice and Future Directions.

[11] Wilson DR (2011). Beyond probabilistic record linkage: Using neural networks and complex features to improve genealogical record linkage.

[12] Kranker K (2018). dtalink - Faster probabilistic record linking and deduplication methods in Stata for large data files.

[13] Uchendu OJ, Nwachokor NF, Eseroghene AI (2020). Study of Adult Injury Fatality in Nigeria.

[14] Roa ECA, Cruz CME, Lazo CAS (2019). Muertes relacionadas a drogas en fallecidos por contexto médico legal, desde el año 2010 al 2018 región de Atacama, Chile; útil herramienta para caracterizar y promover políticas públicas.

[15] Pericolo S (2016). Cocaína e cocaetileno em mortes violentas: levantamento da ocorrência e validação de metodologia para a identificação em humor vítreo com SPME por CG/EM.

[16] Campelo ELC, Caldas ED (2010). Postmortem data related to drug and toxic substance use in the Federal District, Brazil, from 2006 to 2008. Forensic Science International.

[17] Anzillotti L, Marezza F, Calò L, Cucurachi N, Veronesi L, Cecchi R (2019). Toxicological findings: A retrospective overview of medico-legal investigations in Parma (Italy). Journal of Forensic and Legal Medicine.

[18] Coutinho C, Toledo L, Bastos FI (2019). Epidemiologia do uso de substâncias psicoativas no Brasil.

[19] Andrade SM, Cunha MA, Júnior JLP, Maciel AL de S, Santana LSOS, Carvalho RO, Oliveira EH de (2020). Uso crônico e indiscriminado de benzodiazepínicos: uma revisão de literatura. Research, Society and Development.

[20] Fegadolli C, Varela NMD, Carlini EL de A (2019). Uso e abuso de benzodiazepínicos na atenção primária à saúde: práticas profissionais no Brasil e em Cuba. Cad Saúde Pública.

[21] Sales HM (2018). O uso crônico indiscriminado de benzodiazepínicos na atenção básica distrital do município de Nova Russas - CE.

[22] Sanabria E, Cuenca RE, Esteso MÁ, Maldonado M (2021). Benzodiazepines: Their Use either as Essential Medicines or as Toxics Substances. Toxics.

[23] Gondim APS, Nogueira RR, Lima JGB, Lima RAC, Albuquerque PLMM, Veras M do SB, Ferreira MAD, Gondim APS, Nogueira RR, Lima JGB, Lima RAC, Albuquerque PLMM, Veras M do SB, Ferreira MAD (2017). Suicide attempts by exposure to toxic agents registered in a Toxicological Information and Assistance Center in Fortaleza, Ceará, Brazil, 2013. Epidemiologia e Serviços de Saúde.

[24] Cerqueira DRC, Bueno S, Alves PP, Lima RS de, Silva ERA da (2021). Atlas da Violência 2021.

[25] Godoy FJ de, Batista VC, Shibukawa BMC, Oliveira RR de, Marcon SS, Higarashi IH (2021). Mortalidade por causas externas em adolescentes. Revista Enfermagem Atual In Derme.

[26] Moura EC, Gomes R, Falcao MTC, Schwarz E, Neves ACM, Santos W (2015). Desigualdades de gênero na mortalidade por causas externas no Brasil, 2010. Ciência & Saúde Coletiva.

[27] Duke AA, Smith KMZ, Oberleitner LMS, Westphal A, McKee SA (2018). Alcohol, drugs, and violence: A meta-meta-analysis. Psychology of Violence.

[28] Arrantes LL, Pinto FE, Buzin AR, Endringer DC, Andrade TU de, Lenz D (2015). Analysis of the toxicological and epidemiological profile of homicide victims in Vitória, ES, Brazil. Revista Brasileira de Pesquisa em Saúde/Brazilian Journal of Health Research.

[29] Drumond EF, Souza HNF, Hang-Costa TA (2015). Homicides, alcohol and drugs in Belo Horizonte, Minas Gerais State, Brazil, 2000-2009. Epidemiologia e Serviços de Saúde.

[30] Silva BKM, Aguiar ASC de, Almeida PC de, Roscoche KGC, Reis PAM, Martins WA, Moreira JC, Oliveira HS (2021). Análise do perfil de usuários atendidos em um centro de atenção psicossocial álcool e outras drogas. Brazilian Journal of Health Review.

