# Supplementary material for: Sociodemographic factors of violent deaths related to licit or ilicit psychoactive substances: a cross-sectional study, Ceará, Brazil, 2015-2019
Source: Epidemiol Serv Saude. 2025 Jan 27;33:e2024152. doi: 10.1590/S2237-96222024v33e2024152.en (PMC11776068; doi:10.1590/S2237-96222024v33e2024152.en)
Supplement: Translation

# Translation: Fatores sociodemográficos de mortes violentas relacionadas a substâncias psicoativas lícitas ou ilícitas: estudo transversal, Ceará, 2015-2019

**DOI:** 10.1590/S2237-96222024v33e2024152.pt

**Authors:** Wanderley Pinheiro de Holanda, Raimunda Hermelinda Maia Maceno, Maria Augusta Drago Ferreira

**Affiliations:** 1 Perícia Forense do Ceará, Núcleo de Toxicologia Forense, Fortaleza, CE, Brasil; 2 Universidade Federal do Ceará, Departamento de Fisioterapia, Fortaleza, CE, Brasil; 3 Universidade Federal do Ceará, Departamento de Farmácia, Fortaleza, CE, Brasill

**Keywords:** Violência, Toxicologia Forense, Medicina Legal, Drogas de Abuso, Mortalidade, Estudo Transversal

## Abstract

**Objetivo:**

Avaliar a associação entre fatores sociodemográficos, presença de substâncias psicoativas lícitas ou ilícitas e tipos de morte jurídica ocorridas no estado do Ceará

**Métodos:**

Estudo transversal, realizado com base em dados de necropsia e exames toxicológicos em vítimas de casos registrados pela Perícia Forense do Ceará, entre 2015 e 2019.

**Resultados:**

Foram avaliados 4.198 casos; 55,6% foram positivos para substâncias lícitas e/ou ilícitas, com maior frequência de benzodiazepínicos (22,4%) e cocaína (21,7%), no sexo masculino (45,0%), adultos jovens (58,7%), solteiros (49,0%) e com baixa escolaridade (52,8%). Os benzodiazepínicos (29,5%) e antidepressivos tricíclicos (15,6%) foram associados a suicídios; cocaína (28,1%) e maconha (27,1%), a homicídios; e cocaína (28,2%) e antidepressivos tricíclicos (5,9%), às mortes suspeitas.

**Conclusão:**

Fatores sociodemográficos se associaram ao uso de substâncias psicoativas com tipos de morte. Benzodiazepínicos e cocaína foram as substâncias mais frequentes em casos de suicídio e homicídio, respectivamente.

## Contribuições do estudo

**Principais resultados:**

A presença de cocaína e maconha se associaram a maior risco de homicídios, enquanto os benzodiazepínicos e os antidepressivos tricíclicos se associaram a maior risco de suicídios.

**Implicações para os serviços:**

O estudo enfatiza a necessidade de políticas preventivas contra o uso de drogas, bem como tratamento e reinserção de usuários, e medidas de segurança para redução da violência, visando à saúde pública e à justiça social.

**Perspectivas:**

Estudos adicionais são necessários para avaliar a causalidade entre substâncias psicoativas e mortes violentas.

## INTRODUÇÃO

A violência, um problema global, está associada ao uso de substâncias psicoativas.^1^ As causas, influenciadas por múltiplos fatores, são complexas e não totalmente compreendidas.^1,2^ A saúde pública, usando o método científico, busca prevenir a violência através do acúmulo de conhecimentos sobre o tema.^3^

Conhecer as circunstâncias e características das mortes e as substâncias químicas a elas relacionadas é importante para uma melhor avaliação do panorama da mortalidade das vítimas e, por conseguinte, possibilitar a aplicação de políticas e recursos mais eficientes para reduzir tal mortalidade.^4^

A análise da mortalidade e o conhecimento do perfil epidemiológico das vítimas envolvidas em casos de uso de um ou múltiplos agentes químicos, bem como a identificação dos agravos e dos grupos mais suscetíveis, são de fundamental importância para a tomada de ações e desenvolvimento de políticas específicas pelo sistema de saúde.^2^ Entretanto, ainda são limitadas as pesquisas que relacionam o uso de substâncias específicas e os fatores de risco que levam à morte.^4^

Assim, há de se considerar que, além de identificar as substâncias químicas que estão envolvidas nessas mortes, é necessário compreender como o padrão de mortalidade violenta se relaciona com as substâncias.^4,5^ Portanto, mais pesquisas são essenciais para expandir o entendimento e desenvolver estratégias de prevenção e intervenção mais eficazes.^5^

 A violência associada ao uso de substâncias psicoativas requer atenção e estudos contínuos. Pesquisas anteriores já demonstraram essa associação.^4-7^ Dessa forma, a relevância deste estudo reside na verificação da relação entre o uso de substâncias psicoativas e a mortalidade por causas violentas no Ceará. Compreender esse cenário é crucial para a saúde e segurança pública, pois permite a avaliação do panorama da mortalidade e o direcionamento de recursos e políticas públicas mais eficientes para a redução da violência.^4,5^

Nesse sentido, a medicina legal e a toxicologia forense desempenham papel crucial, ao fornecerem informações detalhadas sobre as circunstâncias das mortes. Além disso, ajudam a estabelecer as relações de causa e efeito entre os agentes químicos e as mortes violentas. Como parte da segurança pública, são responsáveis por conduzir investigações forenses e determinar a *causa mortis* em casos de homicídios, suicídios, mortes acidentais ou outras mortes não naturais ou suspeitas.^7-9^

O objetivo do estudo foi avaliar a associação entre fatores sociodemográficos, a presença de substâncias psicoativas lícitas ou ilícitas e tipos de morte jurídica ocorridas no estado do Ceará.

## MÉTODOS

Estudo transversal utilizando técnicas determinística e probabilística de pareamento de registros (*record linkage*), dos casos de morte violentas com suposto envolvimento de substâncias químicas lícitas e/ou ilícitas, registrados pela Perícia Forense do Ceará, no período de 2015 a 2019.

Instituída pela Lei Estadual nº 14.055/2008, a Pericia Forense do Ceará, sediada no município de Fortaleza, é uma entidade técnico-científica subordinada à Secretaria de Segurança Pública, que atua em todo estado do Ceará, e cuja atribuição é a produção de provas de natureza científica nas investigações criminais.^9^

Os dados dos casos analisados no presente estudo foram provenientes dos exames de necropsia feitos no núcleo de Tanatologia Forense da Coordenadoria de Medicina Legal, e os resultados das análises toxicológicas de triagem, realizadas no Núcleo de Toxicologia Forense da Coordenadoria de Análises Laboratoriais Forenses, ambos pertencentes à Pericia Forense do Ceará.

Foram incluídos no estudo os casos que tiveram a análise toxicológica de triagem realizada em amostra de sangue total. Foram excluídos os casos cujos registros indicavam cadáveres em estado de putrefação ou carbonizados, situações em que amostras de sangue da vítima não estavam disponíveis e casos decorrentes da exposição a praguicidas, álcool etílico e substâncias voláteis.

Os dados dos óbitos foram coletados de três sistemas de registro: o Sistema de Gestão Integrada, que operou até 2019; o Sistema Galileu, *software* de gerenciamento de perícia implementado a partir de 2019; e o sistema automatizado Evidence® Randox Laboratories Ltd., que armazena os resultados das análises de triagem toxicológica obtidas pela técnica de imunoensaio.

A vinculação de dados foi realizada utilizando-se pareamento determinístico, probabilístico e junção.^10^

O pareamento determinístico foi feito por meio de um campo ou dado único de identificação (id) comum nos bancos que podem ser ligados.^10^ O pareamento probabilístico foi empregado para identificar dados que se referem à mesma entidade, mesmo que não sejam identificadores únicos. Foram atribuídos pesos (escores), baseados na semelhança entre os valores dos identificadores em potencial. 

Para pareamento e cálculos estatísticos foi utilizado o *software* Stata® (*Statistics/Data Analysis*) BE versão 17. No pareamento determinístico, foram utilizadas as funções “merge” e “merge joinby pairwise”. Os identificadores únicos (id) empregados foram o número da solicitação e a combinação das entidades: código do órgão, número da guia, ano da guia, número do registro, ano do registro e núcleo forense. 

No pareamento probabilístico utilizou-se a função “dtalink” para associar os identificadores, atribuindo-se escores para avaliar a probabilidade de correspondência entre os registros da seguinte forma: nome, ano da guia (+10 para coincidências; -5 para não coincidências) e data de entrada (+5 para dias exatos; -6 para diferenças superiores a 30 dias). 

Para se obter sensibilidade e especificidade na elaboração dos cálculos de probabilidade, estabeleceu-se um critério em que a soma dos escores para a combinação dos pares deveria ser maior ou igual a 34.^12^ Após essa etapa, a consistência do par formado foi avaliada para determinar sua aceitação ou rejeição. 

As variáveis independentes foram categorizadas em sexo (masculino/feminino); idade (≤ 14 anos; entre 15 e 29 anos; entre 30 e 49 anos; e ≥ 50 anos); estado civil (solteiro, separado/divorciado/viúvo, casado/união estável); grau de instrução (não alfabetizado, alfabetizado, 1° grau completo, 2° grau completo, nível superior); ocupação (estudante, trabalhadores dos setores agroindustrial/serviços/comércio, setor público/ciências, aposentado/desempregado); morte jurídica (homicídios, suicídios, acidentais [incluindo acidentes de trânsito], mortes suspeitas [incluindo mortes indeterminadas], outras [afogamentos, eletroplessão etc.]; instrumento/modo da morte. O desfecho foi a detecção de substâncias ilícitas e/ou fármacos nos testes toxicológicos. Ou seja, uma amostra foi considerada positiva (detectada) quando pelo menos uma das substâncias pesquisadas apresentou concentração igual ou superior ao valor de corte em nanogramas por decilitro (ng/mL). 

Os valores de corte para as substâncias ilícitas foram: metanfetamina ≥ 110 ng/mL; anfetamina ≥ 100 ng/mL; 3,4-metilenodioximetanfetamina (MDMA, ecstasy) e cocaína/benzoilecgonina ≥ 50 ng/mL; delta-9-THC (maconha) ≥ 10 ng/mL; fenciclidina ≥ 5 ng/mL. Para os fármacos e classes de fármacos, foram: antidepressivos tricíclicos (TCA) ≥ 60 ng/mL; barbitúricos ≥ 50 ng/mL; benzodiazepínicos, buprenorfina, metadona, oxicodona, opioides, meprobamato e dextrometorfano ≥ 10 ng/mL; opiáceos ≥ 25 ng/mL; tramadol ≥ 5 ng/mL; e fentanil ≥ 2 ng/mL.

Foram aplicados testes estatísticos de Shapiro-Wilk para se verificar a normalidade da distribuição dos dados. A razão de prevalência (RP) foi calculada dividindo-se a prevalência do evento (presença da substância psicoativa) no grupo com mortes relacionadas a drogas/fármacos pela prevalência do mesmo evento em um grupo de referência. O teste exato de Fisher (p-valor bilateral) e o intervalo de confiança de 95% (IC_95_%) foram calculados para se avaliar a significância dessa relação. Resultados com p-valor < 0,05 foram considerados estatisticamente significantes. 

O projeto foi aprovado pelo Comitê de Ética em Pesquisa da Universidade Federal do Ceará, por meio do parecer 5.192.820 de 5/1/2022, certificado de apresentação de apreciação ética 53707521.4.0000.5054.

## RESULTADOS

De 2015 a 2019, foram registrados 5.329 óbitos com suspeita de envolvimento com substâncias químicas psicoativas. Em 4.400 casos foram realizados exames toxicológicos de triagem para detecção de drogas e medicamentos. Após exclusão de 202 registros, 4.198 casos foram incluídos, representando 12,0% dos 34.304 exames de necropsia realizados.

Dos 4.198 casos examinados, 55,6% foram positivos para substâncias lícitas ou ilícitas e 35,9% foram positivos para substâncias ilícitas. A maioria dos indivíduos eram do sexo masculino (81,7%), tinham idade entre 30 e 49 anos (41,3%) ou 15 e 29 anos (27,2%), eram solteiros (67,4%), alfabetizados (59,6%) e trabalhadores dos setores de agricultura, indústria, serviços e comércio (68,4%). A maior frequência de mortes ocorreu em ambiente hospitalar (37,1%), sendo a maioria delas classificadas como mortes suspeitas (53,9%) (Tabela 1). 

**Tabela 1:** Características dos casos com exames toxicológicos *post mortem* realizados, Ceará, 2015-2019

**Características sociodemográficas**	**n**	**%**
**Sexo**	2.347	100,0
Masculino	1.918	81,7
Feminino	429	18,3
**Faixa etária (anos)**	2.212	100,0
0-4	31	1,4
5-14	28	1,3
15-29	601	27,2
30-49	913	41,3
50-59	298	13,5
60-69	178	8,0
> 69	163	7,4
**Estado civil**	1.855	100,0
Solteiro	1.250	67,4
Casado/união estável/viúvo/	436	23,5
separado/divorciado	169	9,1
**Escolaridade**	1.769	100,0
Não alfabetizado	172	9,7
Alfabetizado	1.054	59,6
1º grau completo	127	7,2
2º grau completo	296	16,7
Superior completo	83	4,7
Não informado	37	2,1
**Ocupação**	1.284	100,0
Aposentado/desempregado/estudante	265	20,6
Agropecuário, industriais, serviços e comércio	878	68,4
Orgãos públicos	141	11,0
**Local da morte**	1.215	100,0
Hospital/unidade de pronto atendimento	450	37,1
Zona rural/via pública	175	14,4
Domicílio/unidade prisional	148	13,0
Endereço sem classificação	443	36,5
**Tipo de morte**	2.030	100,0
Suspeita	1.095	53,9
Acidental	438	21,6
Suicídio	237	11,7
Homicídio	203	10,0
Outros	57	2,8
**Modo de morte**	1.609	100,0
Afogamento	59	3,7
Arma branca	6	0,4
Choque elétrico	14	0,9
Enforcamento	55	3,4
Intoxicação	223	13,9
Projétil de arma de fogo	200	12,4
Queda da própria altura	63	3,9
Queda	6	0,4
Outros	983	61,1

Os fármacos e classes de fármacos com maior detecção, excluindo-se os casos oriundos de estabelecimentos de saúde, foram os benzodiazepínicos (22,4%). Entre as substâncias ilícitas, houve maior detecção da cocaína (21,7%), seguida pela maconha (11,6%) (Tabela 2).

**Tabela 2:** Frequência de substâncias detectadas nos casos de mortes violentas, Ceará, 2015-2019

Fármacos ou classes de fármacos detectados	Todos os casos			Origem hospitalar excluída		
	n	Positivos	%	n	Positivos	%
Benzodiazepínicos	4.198	1.074	25,6	3.748	841	22,4
Meprobamato	357	26	7,3	269	19	7,1
Opioides	357	24	6,3	269	16	5,9
Antidepressivos tricíclicos	4.198	190	4,5	4.163	155	3,7
Barbitúricos	4.141	149	3,6	4.141	149	3,6
Oxicodona	357	12	3,4	269	9	3,3
Fentanil	357	29	8,7	269	7	2,8
Tramadol	357	12	3,4	269	5	1,9
Opiáceos	4.137	100	2,4	3.694	64	1,7
Metadona	4.125	38	0,9	3.684	27	0,7
Zolpidem	357	2	0,6	269	2	0,7
Buprenorfina	4.198	8	0,2	3.748	8	0,2
**Substâncias ilícitas**						
Cocaína	4.198	912	21,7			
Maconha	4.198	488	11,6			
Metanfetamina	4.198	292	7,0			
3,4-metilenodioximetanfetamina (MDMA, *ecstasy*)	3.840	127	3,3			
Fenciclidina	4.198	37	0,9			

Observou-se que a detecção de uma única substância, lícita ou ilícita, ocorreu em 29,1% dos casos. Em substâncias ilícitas isoladamente, esse percentual foi de 23,1%. A detecção de múltiplas substâncias apresentou maior associação entre as substâncias ilícitas (12,8%) do que entre as lícitas (8,2%).

Tendo como base o ano de 2015, em 2018 houve detecção significativa de substâncias psicoativas (48,9%), substâncias ilícitas (42,9%) e compostos anfetamínicos (21,6%) (Tabela 3). Maior prevalência foi observada entre homens (45,0%) e jovens de 15-29 anos (32,0%), incluindo substâncias lícitas e ilícitas. Os solteiros (49,0%) apresentaram maior prevalência na detecção de substâncias psicoativas do que os casados/união estável. Indivíduos com 1º grau completo (52,8%) tiveram maior prevalência para substâncias psicoativas, comparados aos não alfabetizados. Entre as ocupações, estudantes se destacaram, com 55,5%. 

**Tabela 3:** Prevalência, razão de prevalência (RP) e intervalo de confiança (IC_95_%) da presença de substâncias psicoativas lícitas e ilícitas, Ceará, 2015-2019

Variáveis	Substâncias lícitas e ilícitas				Substâncias ilícitas			Compostos anfetamínicos			
	n	Positivos (%)	RP (IC _95_ % )	p-valor	Positivos (%)	RP (IC _95_ % )	p-valor	n	Positivos (%)	RP (IC _95_ % )	p-valor
Ano	4.198							3.840			
2015	1.511	533 (35,3)	1,00		461 (30,5)	1,00		1.511	115 (7,6 )	1,00	
2016	963	360 (37.4)	1,06 (0,95;1,18)	0,287	312 (32,4)	1,06 (0,94;1,19)	0,323	960	74 (7,7 )	1,01 (0,76;1,34)	0,93
2017	625	262 (41,9)	1,19 (1,06;1,33)	0,004	219 (35,0)	1,14 (1,01;1,31)	0,041	622	122 (19,6 )	2,58 (2,03;3,27)	<0,001
2018	662	324 (48,9)	1,39 (1,25;1,54)	<0,001	284 (42,9)	1,41 (1,25;1,58)	<0,001	645	139 (21,6 )	2,83 (2,25;3,56)	<0,001
2019	437	190 (43,5)	1,23 (1,09;1,4)	0,002	112 (25,6)	0,84 (0,70;1,0)	0,049	102	9 (8,8 )	1,16 (0,61;2,22)	1,17
**Sexo**	2.347							2.001			
Masculino	1.918	864 (45)	1,11 (1,02;1,32)	<0,001	733 (38,2)	1,23 (1,15;1,31)	<0,001	1.634	232 (14,2)	1,00	
Feminino	429	167 (38,9)	1,00		104 (24,2)	1,00		367	68 (18,5)	1,30 (1,02;1,66)	0,04
**Faixa etária (anos)**	2.213							1.877			
0-14	60	14 (23,3)	1,00		9 (15,0)	1,00		51	6 (11,8 )	0,91 (0,41;1,98)	0,82
15-29	601	353 (58,7)	2,52 (1,58;4,0)	<0,001	322 (53,6)	3,57 (1,95;6,55)	<0,001	531	69 (13,0 )	1,00	
30-49	913	434 (47,5)	2,04 (1,28;3,24)	<0,001	347 (38,0)	2,53 (1,38;4,65)	<0,001	776	122 (15,7 )	1,21 (0,92;1,59)	0,171
> 49	639	172 (26,9)	1,15 (0,72;1,86)	0,549	109 (17,1)	1,13 (0,61;2,12)	0,684	519	93 (17,9 )	1,38 (1,03;1,84)	0,03
**Estado civil**	1.855							1.528			
Solteiro	1.250	613 (49)	1,43 (1,25;1,65)	<0,001	515(41,2)	1,83 (1,52;2,21)	<0,001	1.037	162 (15,6 )	1,07 (0,81;1,40)	0,726
Separado/divorciado/viúvo	169	60 (35,5)	1,04 (0,82;1,32)	0,758	49 (29,0)	1,29 (0,96;1,73)	0,094	134	27 (20,1 )	1,05 (0,79;1,40)	0,158
Casado/união estável	436	149 (34,2)	1,00		98 (22,5)	1,00		357	53 (14,8 )	1,00	
**Escolaridade**	1.732							1.412			
Não alfabetizado	172	57 (33,1)	1,00		39 (22,7)	1,00		137	25 (18,2 )	1,27 (0,86;1,88)	0,237
Alfabetizado	1.054	478 (45,4)	1,37 (1,09;1,7)	0,054	391 (37,1)	1,64 (1,23;2,18)	<0,001	877	126 (14,4 )	1,00	
1° grau completo	127	67 (52,8)	1,60 (1,22;2,08)	0,003	58 (45,7)	2,01 (1,44;2,81)	<0,001	96	14 (14,6 )	1,02 (0,61;1,69)	0,954
2° grau completo	296	138 (46,6)	1,40 (1,10;1,79)	0,033	116 (39,2)	1,72 (1,27;2,35)	<0,001	238	42 (17,6 )	1,23 (0,89;1,69)	0,21
Superior	83	32 (38,6)	1,16 (0,82;1,64)	0,657	21 (25,3)	1,11 (0,70;1,77)	0,624	64	11 (17,2 )	1,2 (0,68;2,10)	0,538
**Ocupação**	1.263							1.144			
Estudante	137	76 (55,5)	1,71 (1,28;2,35)	<0,001	65 (47,4)	1,82 (1,28;2,58)	0,005	127	19 (15,0 )	0,99 (0,63;1,54)	0,959
Agropecuário, industriais, serviços e comércio	893	402 (45)	1,41 (1,07;1,85)	0,008	338 (37,8)	1,45 (1,06;1,99)	0,013	806	122 (15,1 )	1,00	
Orgãos públicos	114	49 (43)	1,34 (0,96;1,88)	0,073	40 (35,1)	1,34 (0,91;1,99)	0,135	102	19 (18,6 )	1,23 (0,79;1,91)	0,360
Aposentado/desempregado	119	38 (31,9)	1,00		31 (26,1)	1,00		109	26 (23,9 )	1,58 (1,09;2,29)	0,02

Tendo por base o ano de 2015, em 2018 houve diferenças significativa na detecção de cocaína (27,0%) e do MDMA (9,6%). A maconha (14,1%) teve maior participação no ano de 2016 em relação a 2019. No que concerne ao sexo, o masculino apresentou prevalências significativa mais altas de cocaína (26,5%) e maconha (12,9%). Na faixa etária de 15 a 29 anos, houve maior detecção de cocaína (38,1%) e maconha (26,0%). Entre as ocupações, tomando-se por base aposentados/desempregados, os trabalhadores do setor público e ciências (31,4%), do setor agroindustrial e serviços (21,9%) e estudantes (7,60%) mostraram prevalências elevadas para cocaína, enquanto estudantes apresentaram maior prevalência para maconha (21,2%) (Tabela 4).

**Tabela 4:** Prevalência, razão de prevalência (RP) e intervalo de confiança (IC_95_%) da presença de cocaína, maconha e *ecstasy*, Ceará, 2015-2019

Variáveis	Cocaína				Maconha			*Ecstasy*			
	n	Positivos (%)	RP (IC _95_ % )	p-valor	Positivos (%)	RP (IC _95_ % )	p-valor	n	Positivos (%)	RP (IC _95_ % )	p-valor
Ano	4.198							3.840			
2015	1.511	302 (20,0)	1,00		202 (13,4)	2,54 (1,67;3,85)	<0,001	1.511	8 (0,5)	1,00	
2016	963	212 (22,0)	1,10 (0,94;1,29)	0,226	136 (14,1)	2,68 (1,75;4,11)	<0,001	960	17 (1,8)	3,34 (1,44;7,71)	0,005
2017	625	124 (19,8)	0,99 (0,82;1,19)	0,938	50 (8,0)	1,52 (0,94;2,45)	0,085	622	38 (6,1)	11,5 (5,41;24,59)	<0,001
2018	662	179 (27,0)	1,35 (1,15;1,59)	<0,001	77 (11,6)	2,21 (1,41;3,46)	<0,001	645	62 (9,6)	18,1 (8,74;37,69)	<0,001
2019	437	95 (21,7)	1,09 (0,87;1,33)	0,423	23 (5,3)	1,00		102	2 (2,0)	3,70 (0,79;17,21)	0,097
**Sexo**	2.347							2.001			
Masculino	1.918	509 (26,5)	2,27 (1,74;2,98)	<0,001	247 (12,9)	3,06 (1,92;4,89)	<0,001	1.634	73 (4,5)	1,00	0,08
Feminino	429	50 (11,7)	1,00		18 (4,2)	1,00		367	25 (6,8)	1,52 (0,98;2,34)	
**Faixa etária (anos)**	2.213							1.877			
0-14	60	3 (5,0)	0,88 (0,28;2,79)	0,083	3 (5,0)	1,88 (0,57;6,23)	0,307	51	4 (7,8)	2,60 (0,90;7,49)	0,082
15-29	601	229 (38,1)	6,76 (4,85;9,44)	<0,001	156 () 26,0	9,75 (5,99;15,89)	<0,001	531	16 (3,0)	1,00	
30-49	913	253 (27,7)	4,91 (3,52;6,87)	<0,001	69 (7,6)	2,84 (1,69;4,78)	<0,001	776	38 (4,9)	1,62 (0,91;2,88)	0,096
> 49	639	36 (5,6)	1,00		109 (17,1)	1,13 (0,61;2,12)	0,684	519	37 (7,1)	2,36 (1,33;4,19)	0,003
**Estado Civil**	1.855							1.528			
Solteiro	1.250	357 (28,6)	2,04 (1,60;2,61)	<0,001	169 (13,5)	3,1 (1,95;4,92)	<0,001	1.037	61 (5,9)	1,31 (0,77;2,24)	0,679
Separado/divorciado/viúvo	169	29 (17,2)	1,23 (0,82;1,84)	0,327	7 (4,1)	0,95 (0,41;2,22)	0,907	134	8 (6,0)	1,33 (0,58;3,04)	0,497
Casado/união estável	436	61 (14,0)	1,00		19 (4,4)	1,00		357	16 (4,5)	1,00	
**Grau de Instrução**	1.732							1.412			
Não alfabetizado	172	17 (9,9)	0,68 (0,34;1,36)	0,284	12 (7,0)	0,96 (0,37;2,48)	0,25	137	11 (8,0)	1,60 (0,84;3,28)	0,350
Alfabetizado	1.054	279 (26,5)	1,83 (1,07;3,11)	0,018	120 (11,4)	1,57 (0,71;3,47)	0,941	877	44 5,0)	1,00	
1° grau completo	127	45 (35,4)	2,45 (1,38;4,35)	0,001	18 (14,2)	1,96 (0,81;4,73)	0,129	96	4 (4,2)	0,83 (0,30;2,26)	0,534
2° grau completo	296	80 (27,0)	1,86 (1,07;3,25)	0,02	28 (9,5)	1,3 (0,56;3,05)	0,531	238	15 (6,3)	1,25 (0,71;2,21)	0,308
Superior	83	12 (14,5)	1,00		6 (7,2)	1,00		64	6 (9,4)	1,86 (0,82;4,21)	0,456
**Ocupação**	1.263							1.144			
Estudante	137	9 (7,6)	4,15 (2,11;8,15)	<0,001	29 (21,2)	6,03 (2,18;16,65)	<0,001	127	7 (5,5)	1,13 (0,52;2,49)	0,745
Agropecuário, industriais, serviços e comércio	893	25 (21,9)	3,54 (1,87;6,69)	0,003	99 (11,1)	3,16 (1,18;8,42)	<0,001	806	39 (4,8)	1,00	
Orgãos públicos	114	43 (31,4)	2,90 (1,41;5,94)	<0,001	4 (3,5)	1,00		102	8 (7,8)	1,62 (0,77;3,37)	0,201
Aposentado/desempregado	119		1,00		2 (1,7)	0,48 (0,09;2,56)	0,389	109	11 (10,0)	2,08 (1,10;3,94)	0,027

A Tabela 5 apresenta a prevalência de substâncias lícitas e ilícitas dos diferentes tipos de morte em relação às mortes acidentais. Os resultados mostram que, nas mortes por suicídio, houve maior prevalência de substâncias lícitas (52,3%), benzodiazepínicos (29,5%) e antidepressivos tricíclicos (15,6%). Nas mortes suspeitas, 45,3% foram positivas para substâncias lícitas, com 15,9% para benzodiazepínicos e 5,9% para antidepressivos tricíclicos. Os óbitos relacionados à detecção de substâncias ilícitas foram significativamente frequentes nos casos dos homicídios (51,7%), seguindo-se as mortes suspeitas (39,8%). A cocaína (28,2%) teve participação significativa nas mortes suspeitas, e, nos homicídios, a cocaína (28,2%), seguida da maconha (27,1%), foram as substâncias mais prevalentes entre as drogas pesquisadas.

**Tabela 5:** Prevalência, razão de prevalência (RP) e intervalo de confiança (IC_95_%) da presença de substâncias lícitas e ilícitas com o tipo de morte, Ceará, 2015-2019

Tipos de morte		Substâncias lícitas				Benzodiazepínicos				Antidepressivos tricíclicos		
	n	Positivos (%)	RP (IC _95_ % )	p-valor		Positivos (%)	RP (IC _95_ % )	p-valor		Positivos (%)	RP (IC _95_ % )	p-valor
Acidental	438	155 (35,4)	1,00			59 (13,5)	1,00			13 (3,0)	1,00	
Homicídio	203	67 33,0)	0,93 (0,74;1,18)	0,555		29 (14,3)	0,93 (0,62;1,40)	0,730		3 (1,5)	0,50 (0,14;1,73)	0,260
Suicídio	237	124 (52,3)	1,48 (1,24;1,76)	<0,001		70 (29,5)	2,11 (1,56;2,84)	<0,001		37 (15,6)	5,26 (2,85;9,70)	<0,001
Suspeita	1094	496 (45,3)	1,28 (1,11;1,48)	<0,001		174 (15,9)	1,22 (0,94;1,60)	0,133		65 (5,9)	2,00 (1,12;3,59)	<0,001
Outros	56	28 (50,0)	1,41 (1,06;1,89)	0,033		12 (21,4)	1,54 (0,90;2,64)	0,127		3 (5,4)	1,80 (0,53;6,14)	0,341
**Tipos de morte**		**Substâncias ilícitas**				**Cocaína**				**Maconha**		
	**n**	**Positivos (%)**	**RP (IC**_95_ **%** **)**	**p-valor**		**Positivos (%)**	**RP (IC**_95_ **%** **)**	**p-valor**		**Positivos (%)**	**RP (IC**_95_ **%** **)**	**p-valor**
Acidental	438	103 (23,5)	1,00			65 (14,8)	1,00			31 (7,1)	1,00	
Homicídio	203	105 (51,7)	2,20 1,77;2,73)	<0,001		57 (28,1)	1,89 (1,38;2,59)	<0,001		55 (27,1)	3,83 (2,55;5,75)	<0,001
Suicídio	237	61 (25,7)	1,09 (0,83;1,44)	0,521		37 (15,6)	1,05 (0,73;1,53)	0,789		13 (5,5)	0,78 (0,41;1,45)	0,424
Suspeita	1094	435 (39,8)	1,69 (1,41;2,03)	<0,001		308 (28,2)	1,90 (1,49;2,42)	<0,001		111 (10,1)	1,43 (0,98;2,10)	0,061
Outros	56	16 (28,6)	1,21 (0,78;1,90)	0,405		11 (19,6)	1,32 (0,74;2,35)	0,348		4 (7,1)	1,01 (0,37;2,75)	0,985

## DISCUSSÃO

No período de 2015 a 2019, observou-se que a prevalência das mortes com a detecção de pelo menos uma substância psicoativa foi elevada. As substâncias mais frequentes detectadas nos exames toxicológicos foram os benzodiazepínicos e a cocaína. A maioria dos indivíduos envolvidos nesses casos eram do sexo masculino, adultos jovens, solteiros, com nível de alfabetização básico e atuava em atividades relacionadas aos setores agropecuário, industrial, de serviços e comércio. Predominaram os tipos de morte suspeita/indeterminada e, como causas da morte, intoxicação e perfuração por arma de fogo. As drogas ilícitas mais prevalentes entre adultos jovens do sexo masculino foram a cocaína e a maconha; entre os óbitos do sexo feminino, os compostos anfetamínicos foram mais frequentes. A cocaína esteve relacionada às mortes suspeitas/indeterminadas, e tanto a maconha quanto a cocaína se vincularam aos casos de homicídio.

Nos casos de mortes avaliados, a maioria dos indivíduos acometidos eram do sexo masculino, adultos jovens, solteiros, com baixa escolaridade. Esses achados corroboram resultados de estudos similares. Pesquisa realizada em Warri, Nigéria, em 2020,^13^ a partir de exames necroscópicos, revelou que indivíduos do sexo masculino (90,5%) e adultos jovens entre 21 e 30 anos (39,8%) foram os mais implicados nessas ocorrências. Em um contexto médico-legal, mortes relacionadas a drogas foram avaliadas em um estudo realizado em Atacama, no Chile, entre 2010 e 2018, revelando que indivíduos envolvidos, em 80,0% dos casos, eram do sexo masculino.^14^ Em estudo de 2016,^15^ foi relatado que, nas cidades de Joinville e Maceió, no Brasil, mortes violentas associadas a cocaína e cocaetileno ocorreram principalmente entre indivíduos do sexo masculino e na faixa etária entre 19 e 50 anos.

Os testes toxicológicos mostraram a presença de pelo menos uma substância psicoativa em mais da metade dos casos de morte violenta avaliados no presente estudo. Em mais de um terço desses casos, foram detectadas drogas ilícitas. Em estudo realizado em Brasília, entre 2006 e 2008,^16^ foi relatado que pelo menos uma substância (droga ou outro agente tóxico) foi detectada em 21,7% dos casos avaliados, com maior frequência em homens jovens, solteiros e com baixo nível de escolaridade. Além disso, outro estudo, realizado em Parma, na Itália, entre 2009 e 2016, em investigações médico-legais, revelou que 71,8% dos homens apresentaram resultados positivos nos testes toxicológicos.^17^

Nos exames realizados no presente estudo, foram detectados com maior frequência os fármacos da classe dos benzodiazepínicos e as drogas ilícitas (cocaína e maconha), em indivíduos do sexo masculino, adultos jovens. Com relação aos fármacos e/ou classes de fármacos, observou-se alta prevalência dos benzodiazepínicos, seguidos pelos compostos anfetamínicos, meprobamato e opioides/opiáceos. Segundo estudo,^18^ no Brasil, no ano de 2015, os medicamentos mais consumidos de forma não prescrita ou diferente da indicação terapêutica foram os benzodiazepínicos (3,9%), opiáceos (2,9%) e anfetamínicos (1,4%). Embora os medicamentos tragam benefícios significativos, o uso inadequado pode resultar em dependência, além de eles poderem até mesmo ser empregados na perpetração de crimes.

Estudos prévios indicam uso indiscriminado de benzodiazepínicos.^19-21^ Apesar de baixa toxicidade, podem provocar efeitos adversos graves, quando utilizados de forma inadequada ou em combinação com outras substâncias.^22^ Um estudo realizado no Centro de Informação e Assistência Toxicológica, em Fortaleza, Ceará, Brasil, no ano de 2013, mostrou que os benzodiazepínicos estavam incluídos entre as principais classes de fármacos implicadas em casos de intoxicação aguda grave, algumas vezes fatais, decorrentes de tentativas de suicídio.^23^

Visto que atuam como depressores do sistema nervoso central, os benzodiazepínicos têm sido administrados a vítimas por meio de alimentos ou bebidas, para cometimento de crimes, como em casos de estupro.^8^ Por essa razão, o uso indiscriminado tem sido associado à violência, criminalidade e/ou mortalidade.^8,19,22^

A violência pode ser exercida de diversas maneiras,^24^ e varia dependendo do sexo da vítima. No sexo masculino, as causas mais comuns de morte violenta são agressões e acidentes de trânsito,^25^ enquanto no sexo feminino o suicídio é mais prevalente.^26^ Existe uma forte relação entre o uso de substâncias psicoativas, lícitas e/ou ilícitas, e a forma de violência cometida. Essa associação tem efeitos em diferentes populações, seja na vitimização ou na perpetração de outros crimes.^27^ Conforme o exposto, o presente estudo revelou que os tipos de substâncias psicoativas e suas combinações variaram de acordo com a idade, o sexo e o tipo de morte violenta. Assim, entre as substâncias ilícitas, a cocaína e a maconha foram detectadas com maior frequência em indivíduos do sexo masculino. Por outro lado, os compostos anfetamínicos (metanfetamina e *ecstasy*) estavam presentes em maior proporção em indivíduos do sexo feminino.

As substâncias lícitas tiveram maior participação nos casos de suicídio, seguidas pelas mortes suspeitas/indeterminadas. Os benzodiazepínicos e antidepressivos tricílicos foram os mais implicados nas mortes por suicídio. Nos casos de homicídio, pelo menos uma substância ilícita estava presente na maioria das vítimas, com maior envolvimento da cocaína e da maconha.

Estudos realizados na América do Sul, na América do Norte e na Austrália, entre 2009 e 2021, apontaram a significativa participação de benzodiazepínicos, sozinhos ou em combinação com outras substâncias, como responsáveis por intoxicações exógenas agudas, decorrentes, muitas vezes, de tentativas de suicídio ou suicídio.^4,6,22,23^ Em 2016, estudo^4^ realizado na Flórida/USA, relatou que as substâncias depressoras do sistema nervoso central como benzodiazepínicos, meprobamato (metabólito do carisoprodol), opiáceos e zolpidem, estão mais associadas a mortes acidentais ou suicídios. Em 2009, um estudo realizado na Austrália investigou a presença de substâncias psicoativas em casos de morte por homicídio e suicídio, e foi detectada a presença de substâncias psicoativas em 65,5% dos casos; múltiplas drogas, em 25,8%; substâncias ilícitas, em 23,9%; e várias drogas ilícitas, em 5,3% dos casos.^6^ Por sua vez, outro artigo^22^ relata que, na Hungria, entre 1990 e 2001, os inibidores seletivos de recaptação de serotonina, barbitúricos e benzodiazepínicos foram as principais substâncias causadoras de mortes por *overdose* em 79,0% dos suicídios.

Os resultados obtidos no presente estudo indicaram uma tendência de aumento da associação entre drogas ilícitas e mortes violentas, especialmente para a cocaína e a maconha. Nos casos de homicídio, as vítimas que utilizaram drogas ilícitas foram, em sua maioria, do sexo masculino, adultos jovens, e as substâncias detectadas com maior frequência foram a cocaína e a maconha. Assim como nos casos de morte suspeita/indeterminada, a cocaína foi a substância detectada com maior frequência. Em pesquisas realizadas no Chile,^14^ entre 2010 e 2018, e no Brasil,^15^ entre 2013 e 2015, foi demonstrado que as substâncias associadas a homicídios foram principalmente a cocaína e a maconha.

Em São Paulo, em 2019, a maioria das vítimas que usavam maconha e cocaína eram do sexo masculino (82,2%), com idade acima de 30 anos (63,0%).^7^ Em outro estudo realizado em Vitória, em 2015, os autores analisaram 150 vítimas de homicídios e constataram que 47,3% delas tiveram testes positivos para maconha, 36,7%, para cocaína, e 20,6%, para ambas as substâncias.^28^ Em Belo Horizonte, ao investigar a associação entre etanol e outras drogas e homicídio, no período de 2000 a 2009, foi detectada a presença de cocaína e de maconha em 25,1% das vítimas.^29^

Neste estudo, as vítimas com exame toxicológico positivo apresentaram um perfil sociodemográfico similar ao dos usuários de drogas em tratamento no Centro de Atenção Psicossocial – Álcool e Drogas em Fortaleza, em 2018. Este perfil inclui indivíduos que iniciaram o uso de substâncias psicoativas na adolescência (63,6%), predominantemente do sexo masculino (84,0%), com média de idade de 38,9 anos, baixa escolaridade (38,2%); e solteiros, separados ou divorciados representando 53,4% dos registros.^30^ As mortes, especialmente entre adultos jovens, do sexo masculino, podem estar relacionadas aos efeitos das substâncias psicoativas, como a desinibição causada pelo etanol e/ou surtos paranoicos pela cocaína, que podem resultar em comportamento violento. Além disso, essas mortes podem estar relacionadas à exposição a locais violentos para a aquisição de drogas e à participação em atividades criminosas.^7^

Por ser um estudo transversal com base em dados secundários, a inferência de causalidade não é possível. Embora sejam identificáveis associações entre a presença de substâncias psicoativas e as mortes violentas, não se pode afirmar que uma causa a outra. Algumas técnicas estatísticas, como modelos de regressão com variáveis de controle, foram empregadas, para minimizar fatores de confusão e reforçar as relações observadas. 

A presença de substâncias psicoativas nas amostras analisadas foi baseada em exames toxicológicos de triagem, de natureza presuntiva. Esses exames requerem confirmação por meio de técnicas analíticas adicionais, como a cromatografia gasosa ou líquida acoplada à espectrometria de massa. A falta de confirmação pode levar a resultados falsos positivos. Os dados referentes ao exame toxicológico foram tratados com cautela e interpretados à luz do conjunto de informações disponíveis. Ressalta-se a importância da realização de análises confirmatórias em estudos futuros, que podem contribuir para a robustez das conclusões.

A incompletude dos dados nos registros originais e as alterações feitas no sistema de registro, no período do estudo, apontam comprometimento com a qualidade e a representatividade das informações coletadas. Foram feitos esforços para garantir a consistência e a integridade dos dados, por meio da verificação cruzada e da correção de inconsistências, sempre que possível.

Os resultados deste estudo demonstraram associação significativa entre diferentes tipos de morte jurídica, características demográficas e o perfil toxicológico, relacionadas às substâncias psicoativas no estado do Ceará. Os dados apontam para a prevalência de substâncias lícitas e/ou ilícitas entre homens jovens, solteiros e com baixa escolaridade. A distribuição dessas substâncias no total de mortes relacionadas mostrou a presença frequente dos benzodiazepínicos e da cocaína. Além disso, foi observada a associação entre drogas ilícitas e mortes por homicídio, especialmente para a cocaína e a maconha, enquanto os benzodiazepínicos e os antidepressivos tricíclicos foram fortemente associados à morte por suicídio. 

A relação entre os tipos de morte jurídica e o uso de substâncias psicoativas indica uma significativa contribuição para os anos de vida perdidos por morte prematura. A identificação do perfil das populações associadas a essas substâncias é crucial para o desenvolvimento de políticas públicas eficazes de prevenção e mitigação das mortes violentas.
